# Route to sustainable lithium-sulfur batteries with high practical capacity through a fluorine free polysulfide catholyte and self-standing Carbon Nanofiber membranes

**DOI:** 10.1038/s41598-017-06593-2

**Published:** 2017-07-24

**Authors:** Du-Hyun Lim, Marco Agostini, Florian Nitze, James Manuel, Jou-Hyeon Ahn, Aleksandar Matic

**Affiliations:** 1 Department of Applied physics, Chalmers University of Technology, 412 96 Göteborg, Sweden; 2 Department of Materials Engineering and Convergence Technology and RIGET, Gyeongsang National University, 501 Jinju-daero, 52828 Jinju, Republic of Korea

## Abstract

We report on a new strategy to improve the capacity, reduce the manufacturing costs and increase the sustainability of Lithium-Sulfur (LiS) batteries. It is based on a semi-liquid cathode composed of a Li_2_S_8_ polysulphide catholyte and a binder-free carbon nanofiber membrane with tailored morphology. The polysulphides in the catholyte have the dual role of active material and providing Li^+^-conduction, i.e. no traditional Li-salt is used in this cell. The cell is able to deliver an areal capacity as high as 7 mAh cm^−2^, twice than that of commercial Lithium-ion batteries (LiBs) and 2–4 times higher than that of state-of-the-art LiS cells. In addition, the battery concept has an improved sustainability from a material point of view by being mainly based on sulfur and carbon and being completely fluorine-free, no fluorinated salt or binders are used, and has potential for upscaling and competitive price. The combination of these properties makes the semi-liquid LiS cell here reported a very promising new concept for practical large-scale energy storage applications.

## Introduction

The recent increased demand for electric vehicles and for the development of renewable energy systems has put focus on the next generation energy storage systems^1, 2^. Lithium-ion batteries (LiBs) represent state-of-the-art of electrochemical energy storage systems with a rather high specific energy density and good cycle life^3, 4^. However, although this technology is adequate for the consumer electronics, it has some serious shortcomings when considering implementation in electric vehicles or as grid energy storage^5^. In particular, limiting factors are related to the active materials in the cathode, commonly based on LiCoO_2_ in commercial LiBs, resulting in high cost and low sustainability due to the low natural abundance and toxicity of the material, but also from an energy density limited by the actual chemistry (e.g. about 140 to 160 Whkg^−1^ in practice)^6, 7^. Thus, the development of alternative chemistries with higher energy density combined with reduced cost (raw materials as well as materials synthesis) and improved sustainability are mandatory steps^1–5^.

Sulfur-based cathodes are characterized by a very higher theoretical energy density, i.e. 2600 Whkg^−1^, and by the use of abundant, non-toxic and cheap raw materials, i.e. sulfur and carbon. These characteristics has put the spotlight on Lithium-Sulfur batteries (LiS) as one of the most promising alternatives to replace the conventional Li-ion technology in high capacity energy storage systems^8, 9^. However, the practical development of commercial cells has been hindered by several issues^10^. The intrinsic low conductivity of sulfur requires the addition of a conductive agent, most commonly carbon, as well as a binder, to prepare a sulfur-carbon composite cathode. The morphology of the composite material has been shown to be of high importance to prevent the dissolution of the sulfur in the electrolyte, resulting in loss of active material and “shuttle reactions” at the lithium anode surface^11^, and has been addressed by developing mesoporous or microporous carbon structures to encapsulate the sulfur^12–15^. However, the use of carbon, or other conductive matrices, and binders reduces the amount of active material in the electrode, i.e. sulfur, commonly down to 30–40%, and consequently decreases the practical capacity of the LiS battery. Thus, the limited loading of active material in a LiS-cell, and the resulting areal capacity (mAh/cm^2^), is usually lower than that of conventional LiBs even though the specific capacity, reflecting the active material utilization (mAh/g), is high.

In this work, we present a route to improve the practical capacity, safety, and sustainability of LiS-batteries. The approach is based on a semi-liquid LiS-cell utilising a binder-free Carbon Nanofiber (CNF) membrane and a catholyte, formed by adding Lithium-polysulphide to an organic solvent. A novelty of our approach is that the polysulphides play the dual role of active material and Li^+^-conducting agent, i.e. no traditional, commonly fluorinated, Li-salt is added to the electrolyte solution.

The use of polysulphides in the electrolyte has previously been shown to improve both capacity and cycling stability of LiS-cells^16^. The improved performance has been related to preventing active material dissolution from solid cathodes, thanks to a buffering effect, as well as the stabilization of the solid electrolyte interphase (SEI) on the Li-anode^17–22^. Recently, the self-healing effect of dissolved polysulfides on sulfur-nanoparticles has been demonstrated^23^. However, most of the approaches used so far still suffer from issues related to too low practical capacity, high cost of the material used as support for the electrochemical process and/or from sustainability and safety issues due to the use of fluorinated binders and lithium salts^24–26^. In our approach, there are no fluorinated components in neither the catholyte or in the CNF membrane used to support the electrochemical reactions on the cathode side. In addition, the CNF membrane is prepared following a rather straightforward route by electrospinning of a precursor polymer matrix followed by carbonization to create a self-standing and highly conducting membrane, omitting the need for binders or current collectors. We demonstrate that by tailoring the morphology of the CNF membrane, by increasing porosity and pore size distribution, we can build a semi-liquid LiS-cell that can deliver a capacity as high as 7 mAh cm^−2^ (corresponding to a specific capacity of 1200 mAh g_s_^−1^ and with a sulfur loading of 6.5 mg cm^−2^), a value twice as high as that of commercial Lithium-Batteries (LiBs) and 2–3 times higher than Li-S systems commonly reported as state-of-the-art^17–27^.

## Results

### Concept of Polysulfide as Li^+^-conducting medium

Despite the fact that during the past five years the use of polysulphide based catholytes for LiS-cells has been investigated by several groups^18–26^, the consideration that Li_2_S_x_ could be also play the role of the Li-salt in the electrolyte, i.e. provide Li^+^ conduction, has to the best of our knowledge not been considered. In this work we selected the long-chain Li_2_S_8_ polysulfide as the base of the catholyte due to its high solubility in organic solvents and due to the high theoretical specific capacity^18–26^. The basic reaction steps in a Li/S cell and the corresponding discharge profile are shown in the supplementary information section (SI), Figure S1. In general, the starting point is elemental sulfur with theoretical specific capacity of 1675 mAh g_s_^−1^, but starting from Li_2_S_8_, which is the second step in the general reaction scheme, one still retains 1477 mAh g_s_^−1^.

**Figure 1:**
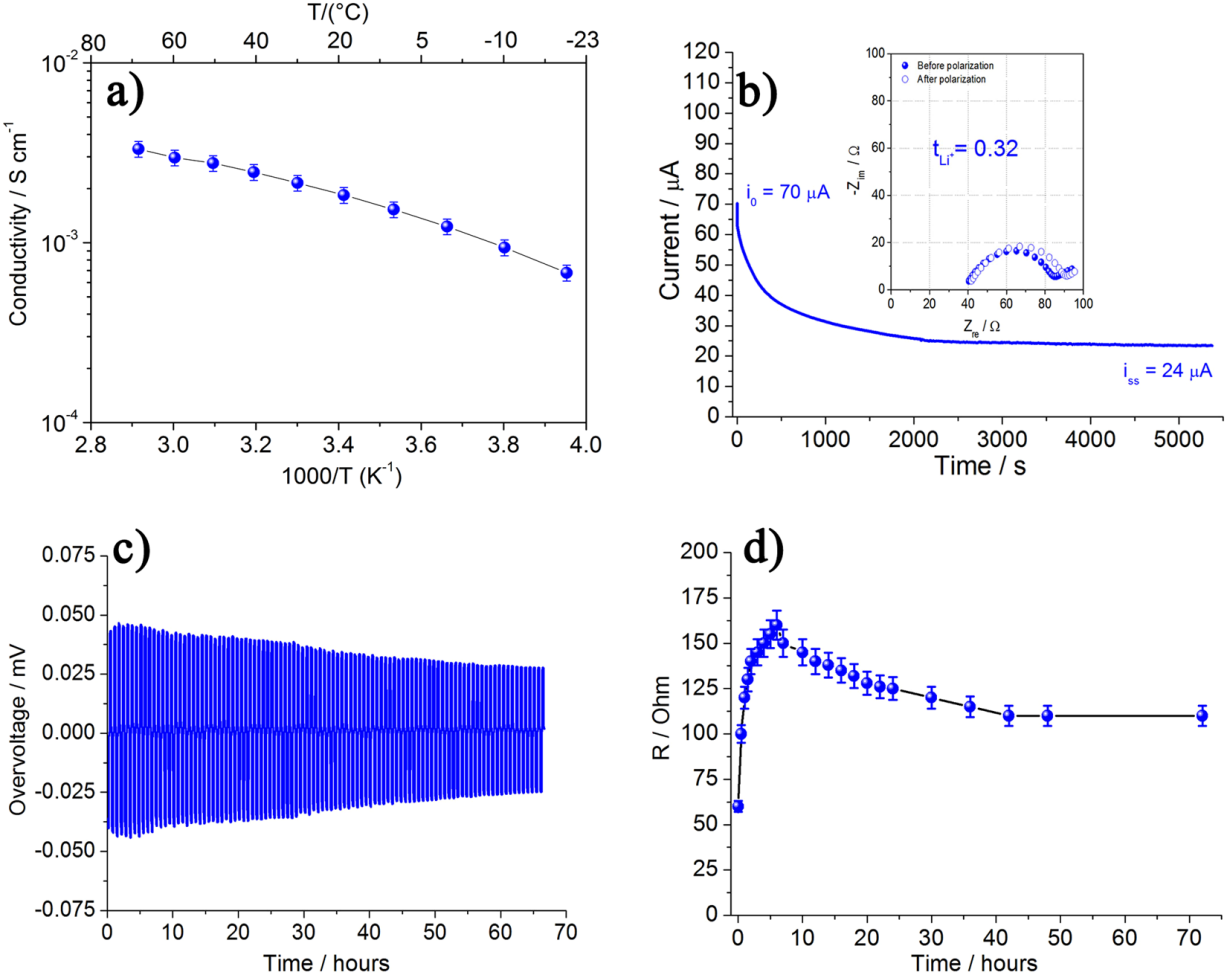
(**a**) Temperature dependence of the conductivity of the catholyte, TEGDME_3_-DOL_7_ 0.5 M Li_2_S_8_, in the temperature range −20 °C to 70 °C. (**b**) Time evolution of the current in symmetric, Li/TEGDME_3_-DOL_7_ 0.5 M Li_2_S_8_/Li, cells following a DC polarization of 30 mV. Inset shows the Nyquist plots before and after the cell polarization. (**c**) Lithium stripping-deposition overvoltage of the symmetric cell cycled at a current density of 0.l mA/cm^2^ with LiNO_3_ added to the catholyte (see text). (**d**) Time evolution of the overall cell resistance during the stripping-deposition experiment in Fig. 1c.

In Fig. 1 we report on the temperature dependence of the ionic conductivity of the catholyte, formed by dissolving 0.5 M Li_2_S_8_ in TEGDME_3_-DOL_7_ (tetraethylene glycol dimethyl ether and dioxolane). The data shows that the conductivity is higher than 10^−3^ S cm^−1^ between −20 and 70 °C. This compares well with conductivity values found for common liquid electrolyte solutions using conventional Li-salts, such as LiTFSI or LiCF_3_SO_3_^27^, and is viable for practical applications. Additionally, Figure S2 shows the conductivity for a polysulphide concentration in the electrolyte solution corresponding to the concentration at the end of the discharge confirming that the value is still in the range necessary for batteries applications. To characterize the lithium-ion conduction in the catholyte we have measured the Li^+^ transference number (t_Li_^+^) according to the Bruce-Vincent method^28^. The t_Li_^+^ is calculated from the time evolution of the overall resistance of a symmetrical cell (Li/catholyte/Li) and from the impedance before and after cell polarization and this data is reported in Fig. 1b. From this data we determine a Li^+^ transference number t_Li_^+^ = 0.32, i.e. a bit lower than what is reported for classic electrolytes based on organic solvents and fluorinated Li-salts, but still in a range suitable for electrochemical applications^27^.

In our LiS-cell we are using Li-metal as the anode, thus we have to ensure that the catholyte is stable towards Li-metal. It is well known that the addition of LiNO_3_ to an electrolyte solution favours the formation of a protective layer on the Li-metal surface, mitigating both the lithium-polysulphide reactivity and the shuttle effects^20, 21^. Following this approach 0.1 mole of LiNO_3_ has been added to the TEGDME_3_-DOL_7_ 0.5 M Li_2_S_8_ catholyte and Fig. 1c reports the lithium plating-stripping profiles of a symmetrical cell using this solution. This measurement, together with electrochemical impedance spectroscopy, can be used to evaluate the stability of the film formed on the Li-anode and related surface corrosion^21, 26^. The data shows that initially the polarization increases slightly and then decreases to a stable level after 60 hours. This result and the fact that no voltage spikes appear during the process reveal that the catholyte has a good compatibility towards Li-metal, stabilizing the SEI film and preventing corrosion. An additional stripping/deposition measurement is performed at higher current density (1 mAcm^−2^) with different step conditions (1 h charge/1 h discharge) to further confirm the stability of the electrolyte/Li interphase, see Figure S3 in the SI section. The favourable behaviour is also confirmed by the time evolution of the overall resistance of the symmetrical cell, see Fig. 1d, measured by impedance spectroscopy. The resistance increases initially followed by a decrease that can be ascribed to a partial dissolution of the SEI film. Finally, the cell resistance is stabilized around 125 Ω after 60 hours, thus matching the response in the stripping-deposition experiment.

### Characteristics of the self-standing Carbon-Nanofiber membranes

**Figure 2:**
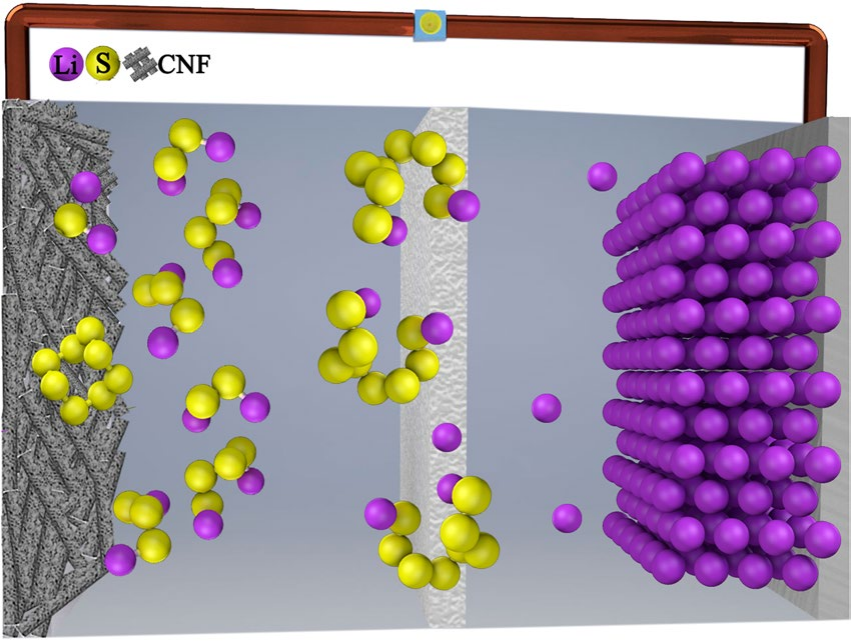
Schematic illustration of the Li-S cell configuration based on semi-liquid and fluorine-free catholyte, binder free and self-standing carbon nanofibers (CNF) and lithium metal anode.

In a semi-liquid battery, a solid working electrode matrix supports the reduction-oxidation process of the active material in the catholyte upon galvanostatic cycling. Our strategy is to use a self-standing CNF membrane and the key is to tune the morphology in order to optimize the use of active material in the electrochemical process. An illustration of the Li-S cell configuration based on semi-liquid and fluorine free catholyte (Li_2_S_8_), binder free and self-standing CNF and lithium metal anode is reported in Fig. 2.

**Figure 3:**
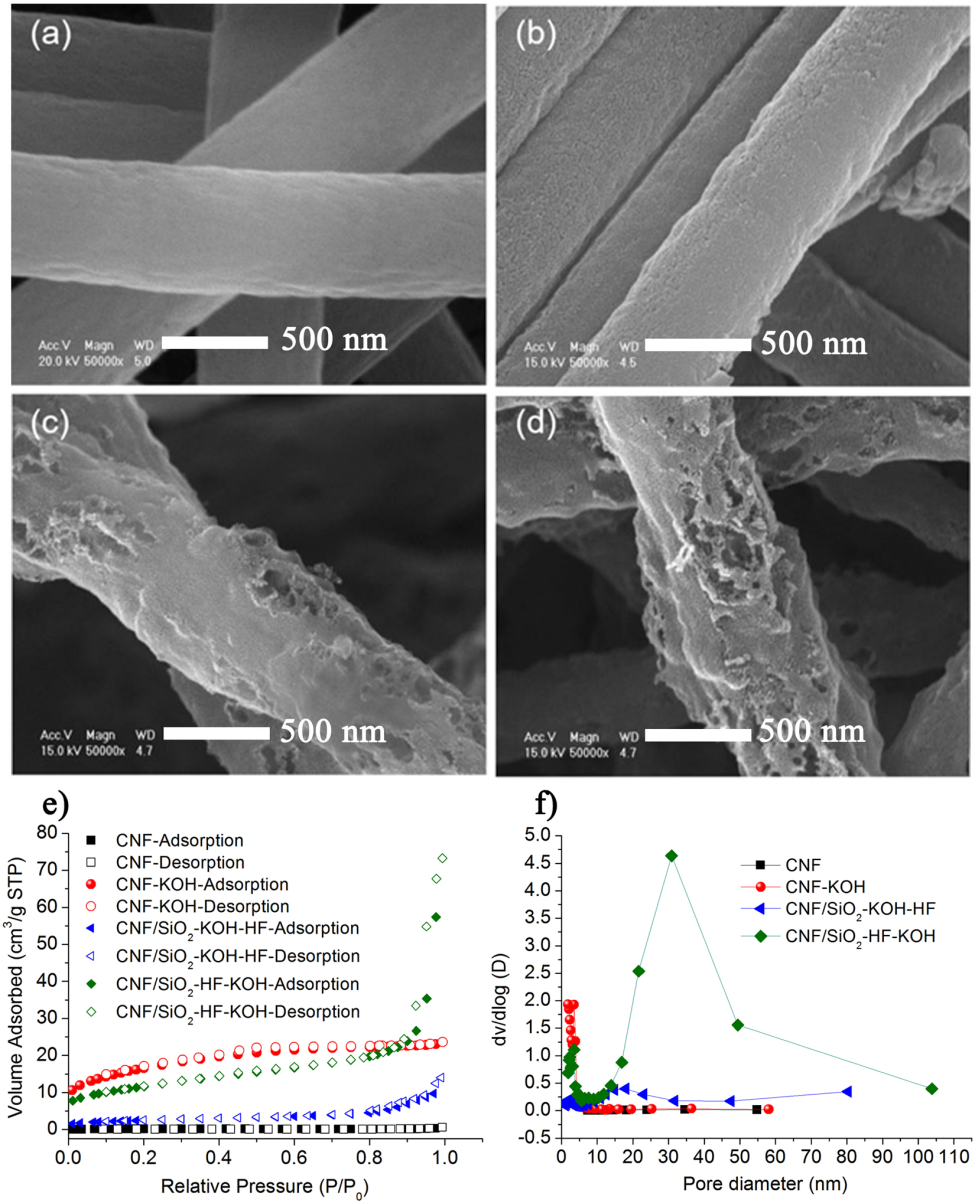
SEM images of the (**a**) pristine CNF, (**b**) CNF-KOH, (**c**) CNF/SiO_2_-KOH-HF and (**d**) CNF/SiO_2_-HF-KOH membranes, respectively. (**e**) BET N_2_ adsorption-desorption isotherms and (**f**) pore size distribution determined from the BET measurements.

The morphology of the membrane has been modified using two routes, by chemical activation in a KOH solution and by inclusion of SiO_2_ nano-particles in the fibers during the preparation of the precursor membrane and the removal of these by HF treatment, see Methods section. Both these routes introduce pores in the fibers and increase the surface area of the material. From Scanning Electron Microscopy (SEM) images, Fig. 3a–d, we can conclude that the pristine CNF membrane is built up of fibers with an average diameter of 500 nm with a smooth surface, whereas the KOH activation as well as the removal of the SiO_2_ nanoparticles by HF etching result in a much rougher surface appearance.

The morphology of the membranes was further characterized by the Brunauer-Emmet-Teller (BET) method in terms of surface area and pore size distribution and the results are reported in Fig. 3e–f and in Table S1 in the SI section. In accordance with the smooth appearance in the SEM image, the pristine CNF membrane has a low surface area, 8 m^2^/g and meso-pores with a size of 34.6 nm on average. After KOH activation the surface area increases dramatically to a value of 1313 m^2^/g due to the formation of micro-pores with an average size around 3.5 nm. In the case of the CNF membrane containing SiO_2_ nanoparticles two different routes can be considered to tune the morphology. By first activating the CNF/SiO_2_ membrane in KOH and then removing the nanoparticles by HF (samples labelled CNF/SiO_2_-KOH-HF) a relatively low surface area, 200 m^2^/g, and a wide pore size distribution, between 2 to 18 nm, is obtained. We attribute the low surface area to the final HF treatment which removes the SiO_2_ nanoparticles but also etches away some of the micro-pores created by KOH. Changing the order of these two steps, first removing the SiO_2_ nanoparticles by HF and then activating the carbon fibers by KOH, results in a high surface area, 924 m^2^/g, but also in the formation of both micro-pores (around 3.5 nm) and meso-pores (around 30 nm).

### Investigation of the electrochemical performance and reaction mechanism

**Figure 4:**
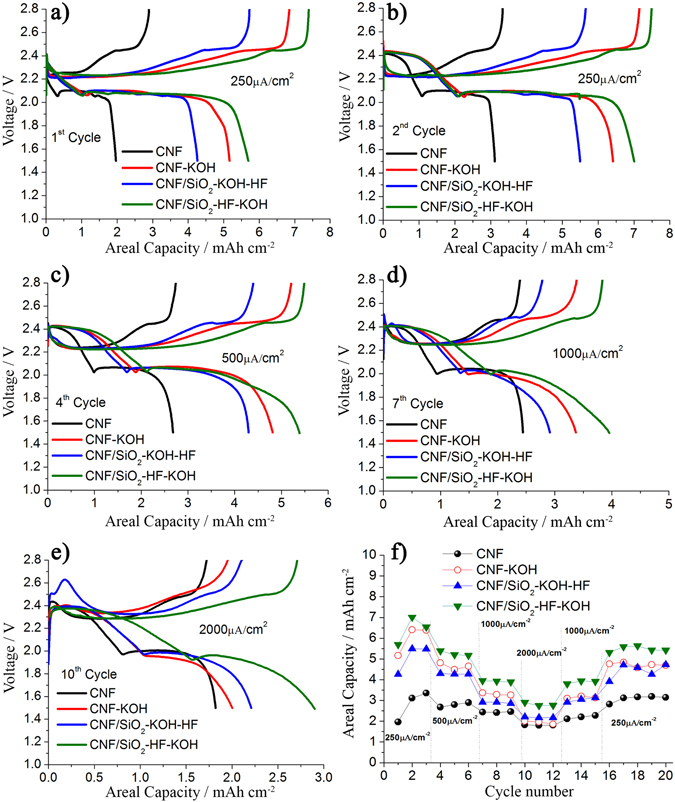
Discharge-charge voltage profiles at different current densities of LiS-cells with different CNF membranes (see caption in the graphics) (**a**) 1^st^ cycle at 250 μA cm^−2^, (**b**) 2^nd^ cycle at 250 μA cm^−2^, (**c**) 4^th^ cycle at 500 μA cm^−2^, (**d**) 7^th^ cycle at 1000 μA cm^−2^ and (**e**) 10^th^ cycle at 2000 μA cm^−2^. (**f**) Discharge areal capacity at the various current densities.

To evaluate the performance of the LiS-cell concept with a polysulphide catholyte acting as both active material and Li-salt and a CNF membrane as support for the electrochemical process, galvanostatic cycling tests were performed. Figure 4a–e show the discharge/charge profiles at different current densities (discharge rates) for cells utilising CNF membranes with different morphologies. The measurements are performed by changing the current density each 3 cycles, from 250 μAcm^−2^ to 2000 μAcm^−2^ (corresponding to a discharge rate, or C-rate, of 0.026 and 0.2 C respectively) and back. During the 1^st^ discharge, Fig. 4a, one can only observe one plateau, around 2.1 V, whereas in the subsequent cycles two plateaus are observed, at 2.4 and 2.1 V respectively. This is a consequence of the fact that the first discharge starts from Li_2_S_8_ and thus the first conversion step, from S_8_ to Li_2_S_8_ and related to the plateau at 2.4 V, is absent. In the subsequent first charge, there is a full reduction to S_8_, thus the first plateau at 2.4 V is observed in the second cycle and the delivered capacity of the cell is also higher.

In the second discharge the cell with the pristine CNF membrane delivers an areal capacity of 3 mAh cm^−2^, corresponding to a specific capacity of 460 mAh g_s_^−1^, see Fig. 4b. The areal capacity is in this case already quite good compared to traditional solid state cathodes for LiS-batteries^29, 30^, but the active material utilization is poor. In contrast, in the cells where the morphology of the CNF membrane has been modified by activation in KOH and removal of the SiO_2_-nanoparticles by HF the capacity is considerably higher, between 5.5 and 7 mAh cm^−2^, with corresponding specific capacities between 840–1100 mAh g_s_^−1^ which is up to 75% of the theoretical capacity of the catholyte. The correlation between the delivered capacity and the pores volume of the CNFs membranes is further demonstrated by Figure S4, reported in the SI section.

The catholyte/CNF cells also show high capacity at higher current densities. In Fig. 4f the current density dependence (or rate dependence) for the cells with different CNF membranes is shown. As expected the capacity decreases with increasing current density, but even when the current density is increased almost 10 times, to 2000 μA cm^−2^, the capacity is still in the range 2–3 mAh/cm^2^. Furthermore, the cells recover 85% of the initial capacity when the current density is lowered back to the initial value, 250 μA cm^−2^, thus confirming a good capacity retention and cycle life.

**Figure 5:**
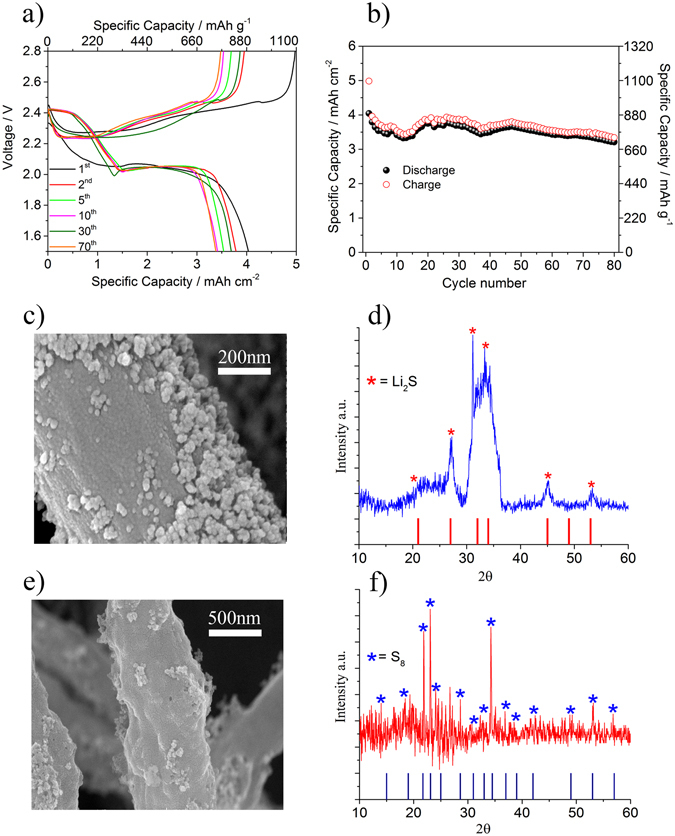
(**a**) Discharge-charge voltage profiles at 1000 μA cm^−2^ for the LiS-cells using the CNF/SiO_2_-HF-KOH membranes and (**b**) capacity as a function of cycle number. (**c**) SEM image and (**d**) XRD- pattern of the CNF/SiO_2_-HF-KOH membrane after complete discharge. (**e**) SEM image and (**d**) XRD- pattern of the CNF/SiO_2_-HF-KOH membrane after charge.

In order to demonstrate the stability of the semi liquid Li/S system, prolonged cycling test has been performed using the most performing membrane, i.e. CNF/SiO_2_-HF-KOH, using a current rate of 1000 μA cm^−2^ (about C/10). Figure 5 shows the discharge/charge voltage profiles and the capacity as a function of cycle number. The cell is able to deliver an areal capacity as high as 4 mAh cm^−2^ at cycle 40^th^ and 3.5 mAh cm^−2^ after 80 cycles with a Coulombic efficiency ranging between 99% to 98%, thus confirming the good cycling performance.

To better understand the electrochemical mechanism of the CNFs/catholyte system SEM and XRD measurements have been performed on the CNF/SiO_2_-HF-KOH membrane after discharge and charge. Figure 5c shows the SEM image of the membrane after full discharge. The image shows that material is deposited on the surface of the fibers, which are almost completely covered by the deposit, during discharge.

From the analysis of the XRD pattern in Fig. 5d it is clear that the deposits are Li_2_S (JCPDS#772145). The peaks in the XRD pattern are rather broad indicating that the deposit is built up of small crystallites on the surface of the CNF. The SEM image after the subsequent charge, Fig. 5e, also reveals presence of particles on the surface of the fibers, however the surface coverage is much smaller. The XRD-analysis reveals that this is related to the formation of S_8_ (JCPDS#850799). The low coverage of sulfur on the surface of the fibers does not mean that only a small fraction of the active material has been fully converted during charge, but rather that most of the sulfur is distributed in the pores of the fibers, as confirmed also by TGA analysis, Figure S5. The full conversion to sulfur during charge is also confirmed by immersion of the charged cathode in ethanol, see Figure S6 in the SI section.

## Discussion

From the data presented above it is clear that the new concept presented here is a promising route to LiS-cells with a high practical capacity. The results show that the capacity of the LiS-cell based on this concept delivers an areal capacity of up to 7 mAh cm^−2^ which is 2 times higher than that of the commercial LiBs and 2–3 times higher than LiS cells based on state-of-the-art solid-state cathodes^18–27, 29–31^. A key for the performance is the morphology of the CNF membrane supporting the electrochemical reactions in the catholyte. Correlating the cell performance to the morphology we can conclude that both the surface area and the pore size distribution is of importance. In particular, the presence of both micro- and meso-pores is beneficial both for a high capacity and for good rate capability. The membrane with the highest surface area (CNF-KOH) does not provide the highest capacity, but here there are only micro-pores (3 nm) present and they might not be fully accessible for the active material in the catholyte. The presence of only micro-pores also decreases the rate capability. The cell with the CNF/KOH membrane delivers a high capacity at lower current densities but at the highest current density (2000 μA cm^−2^) it decreases dramatically and is lower than the capacity of the cell with the CNF/SiO_2_-KOH-HF membrane which has a rather low surface area but both micro- and meso-pores. The relevance of the presence micro- and meso-pores relates to the actual reaction mechanism where during discharge the final reaction involves the deposition of Li_2_S on the surface (where meso-pores are important) whereas during charge a large amount of sulfur goes into the fibers and the micro-pores. Thus, surface area is important during discharge whereas pore volume is the key parameter during charge.

A second central part of our work is the use of a catholyte where the polysulphides have the dual roles as carrier of the active material and as Li-salt providing Li-ion conduction. The conductivity of the catholyte and the Li^+^ transference number are both high enough to provide efficient transport of Li-ions. One can note that even in the case of the cell with the highest capacity, delivering 7 mAh/cm^2^, the active material utilization is 80%. This is of importance since this implies that 20% of the polysulphides are retained in the electrolyte after full discharge and can act as Li-salt supporting the Li-ion transport. Figure S2 shows the conductivity for a polysulphide concentration in the electrolyte solution corresponding to the concentration at the end of the discharge confirming that the value is still in the range necessary for Li-battery applications, Furthermore, the ability to create a stable interphase on the Li-anode as shown by the stripping-deposition experiments is a key to cycling stability of the cell.

The practical capacity of this concept, combined with stable cycling and good rate capability has promises for bringing LiS-batteries closer to applications. An additional factor in favour is also the improved sustainability and safety aspects by the absence of fluorinated components. The rather straightforward preparation of the CNF membrane through electrospinning has potential for upscaling and low cost which are also crucial when introducing a new concept for large scale applications.

## Methods

### Preparation of CNF membranes and stabilization

The CNF membranes where prepared by carbonization of precursor polyacrylonitrile (PAN) nanofiber membranes prepared by electrospinning. In a typical run, a 12 weight % solution was prepared by mixing PAN (M_w_ = 15000, Aldrich) in N,N-dimethylformamide (DMF, Aldrich) by ball milling (1000 rpm) at room temperature for 1 h and subsequently degassing to remove air bubbles. To obtain membranes with silica nanoparticles in the fibers 20 weight % SiO_2_ (10–20 nm, Aldrich) was added to the solution. The solution was fed through a syringe pump (KD Scientific, Model 210) at a constant flow rate of 0.1 ml/min, connected to a needle with a diameter of 0.6 mm. A DC voltage of 20 kV was applied and the distance between the tip of the syringe and the collector, an aluminium foil fixed on a grounded stainless steel rotating drum at 150 rpm, was 20 cm^32^. The thickness of the as prepared PAN membranes was 150–200 µm. The membranes were dried under vacuum at 70 °C for 12 h before stabilization at 250 °C for 1 h. Subsequently the membranes were carbonized by heating from room temperature to 1000 °C, temperature rate 10 °C min^−1^, and kept at 1000 °C for 1 h under nitrogen flow (800 mL min^−1^). The carbon nanofiber membranes obtained from this procedure are labelled as CNF and CNF/SiO_2_ respectively. The morphology of the CNF and CNF/SiO_2_ membranes was modified following three different protocols:

1. The CNF membranes (3 g) were activated with KOH (8 M, KOH solution, 200 ml) in a lab shaker, 50 rpm, at 50 °C for 4 h, and without shaking for 20 h. The activated samples were wiped dry and then dried at 70 °C for 12 h. To remove residual potassium the membranes heated in a tube furnace under nitrogen flow (N2, 99.99%) of 800 mL min^−1^ from room temperature to 750 °C (10 °C min^−1^), kept at 750 °C for 3 h, and finally heated to 1000 °C. The samples were cooled to room temperature, washed with water until the pH became 7.0, dried at 70 °C for 12 h in air and 24 h under vacuum at 70 °C^33^. These samples are labelled as CNF-KOH
2. CNF/SiO_2_ membranes were first activated with KOH following route (1) and then treated with an HF solution (50 weight %) for 24 h in order to remove the SiO_2_ nanoparticles. After HF etching the samples were washed with water until the pH became 7, dried in air at 70 °C for 12 h and under vacuum for 24 h^34^. These samples are labelled CNF/SiO_2_-KOH-HF.
3. CNF/SiO_2_ membranes were first treated with the HF solution, as in route (2), and then activated with KOH following route (1). These samples are labelled CNF/ SiO_2_-HF-KOH.

The areal loading of the CNFs was between 2.0 and 2.5 mg/cm^2^.

### Preparation of catholyte

The Li_2_S_8_–catholyte was prepared by mixing lithium sulphide (Li_2_S, Aldrich) and sulfur (Aldrich) in a 1:7 molar ratio in tetraethylene glycol dimethyl ether (TEGDME, Aldrich) at room temperature and stirring the solution for 24 h. The solution was then mixed with dioxolane (DOL, Aldrich) with 3/7 w/w ratio. Finally, 0.1 mole of LiNO_3_ (Aldrich) was added to 1 liter of TEGDME_3_-DOL_7_ 0.5 M Li_2_S_8_ and stirred for 24 h. The final composition of the catholyte was TEGDME_3_-DOL_7_ 0.5 M Li_2_S_8_-0.1 M LiNO_3_.

### Materials characterization

A stainless steel cell with Teflon O-ring spacers was employed to measure the ionic conductivity of the TEGDME_3_-DOL_7_ 0.5 M Li_2_S_8_ catholyte from −20 °C to 70 °C. The thickness and the diameter were of 1 mm and 1 cm respectively. The measurements were performed on a Novocontrol broadband dielectric spectrometer in the frequency range n 0.01 Hz–1 MHz. The Lithium transference number was obtained following the Bruce-Vincent method^28^, by applying DC polarization of 30 mV to a symmetrical (Li/catholyte/Li) cell and measuring the resistance by Electrochemical Impedance Spectroscopy (EIS) applying 10 mV AC amplitude in the frequency range from 500 KHz to 100 mHz^32^. The stripping-deposition measurement was performed at a current density of 0.1 mA cm^−2^ (15 min charge, 15 min discharge and 5 min open circuit for each cycle). The overall resistance of the Li-cell used for the stripping-deposition was measured by EIS applying 10 mV AC amplitude in the frequency range between 500 KHz to 100 mHz. All the above tests were carried out on a VSP Biologic instrument. The morphology of the CNF membranes was investigated by using field emission scanning electron microscopy (FE-SEM, JEOL JSM 5600) working at 15 kV. The specific surface area, the pores volume, and the pores size distribution were measured with a Brunauer–Emmett–Teller analyzer (BET, ASAP 2010). The XRD measurements have been performed on a Bruker D8 Advance instrument, using a A100-B37 holder (Bruker) closed system, in order to avoid exposure to air during the experiment. Thermogravimetric analysis was performed using TGA Q50, TA Instruments. The sample was kept in an aluminium pan and heated from 25 °C to 500 °C using a rate of 10 °C/min.

### Electrochemical characterization

The electrochemical tests of the Li/ TEGDME_3_-DOL_7_ 0.5 M Li_2_S_8_-0.1 M LiNO_3_/CNF cells were performed on Swagelok-type configuration, with a Celgard 2400 separator, by using an automatic galvanostatic charge-discharge WBCS3000 battery cycler (WonA Tech. Co.) in the voltage range between 1.5–2.8 V. Current densities of 250, 500, 1000 and 2000 μA cm^−2^ (corresponding to the C-rates 0.025, 0.05, 0.1 and 0.2 C, respectively). All materials preparation and the cell assembly were performed in inert atmosphere (H_2_O and O_2_ levels < 10 ppm). The amount of the electrolyte in the Swagelok-type configuration was of 38 μl/cm^2^ (about 4.9 mg/cm^2^ of sulphur), i.e. 52 μl using a 14 mm separator, 12 mm CNF counter electrode/support and 14 mm Lithium foil as anode.

## Electronic supplementary material

Supplementary Information
