# Supplementary material for: Route to sustainable lithium-sulfur batteries with high practical capacity through a fluorine free polysulfide catholyte and self-standing Carbon Nanofiber membranes
Source: Sci Rep. 2017 Jul 24;7:6327. doi: 10.1038/s41598-017-06593-2 (PMC5524792; doi:10.1038/s41598-017-06593-2)
Supplement: Supplementary file 1 — Supplementary Information [file 41598_2017_6593_MOESM1_ESM.pdf]

Route to sustainable lithium-sulphur batteries with high practical capacity through a fluorine free polysulfide catholyte and self-standing Carbon Nanofiber membranes.

Du-Hyun Lim[a,b], Marco Agostini[a], Florian Nitze[a], James Manuel[b], Jou-Hyeon Ahn[b,*], Aleksandar Matic[a,*]

[a] *Department of Applied physics, Chalmers University of Technology, 412 96 Göteborg, Sweden*

[b] *Department of Chemical Engineering and Research Institute for Green Energy Convergence Technology, Gyeongsang National University, 900 Gajwa-dong, Jinju 660-701, Republic of Korea*

[*]Corresponding authors:matic@chalmers.se; jhahn@gnu.ac.kr

Supplementary Information

The basic reaction steps, and the corresponding discharge curve, in a Li/S-cell are shown in Figure S1. This full electrochemical reaction occurs in a series of steps involving the formation of a polysulphides (Li_2S_x, x=2-8), with different contributions to the total specific capacity. In our case the starting point is Li_2S_8 and the theoretical specific capacity of the cell is thus reduced by 12% to 1477 mAh g^{-1}.

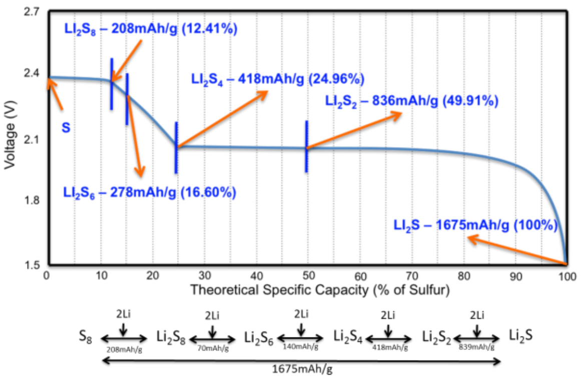

Figure S1. Schematic illustration of a discharge profile of Li-S battery and the reaction mechanism.

Ionic conductivity has been measured for a polysulphide concentration in the electrolyte solution corresponding to the concentration at the end of the discharge, i.e. around 0.1M Li_2S_8. In Figure S2 the conductivity at the two different concentrations are compared. As expected the ionic conductivity decreases when the concentration of polysulphides is reduced, from 10^{-3} S/cm to 10^{-4} S/cm. This value is still in the range of Li-ion batteries applications and provides Li^+ ion conduction.

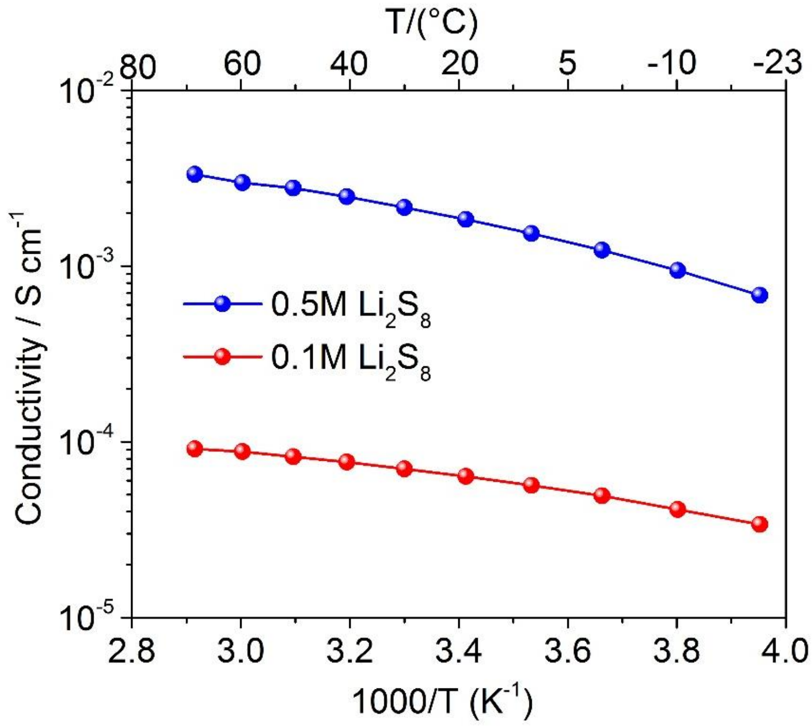

Figure S2. Comparison of the ionic conductivity between the 0.5M Li_2S_8 (blue dots) and 0.1M (red dots) $TEGDME_3$-DOL_7 solutions.

To further demonstrate the stability of our electrolytic system we performed an additional stripping deposition measurement using a higher current, 1 mA/cm^2, and 1hr charge/discharge step. Figure S3 reported in the Supplementary Information section shows a stable overvoltage of about 70 mV even after more than 200h of Li plating/stripping, confirming a good stability of the electrolyte/Li interphase.

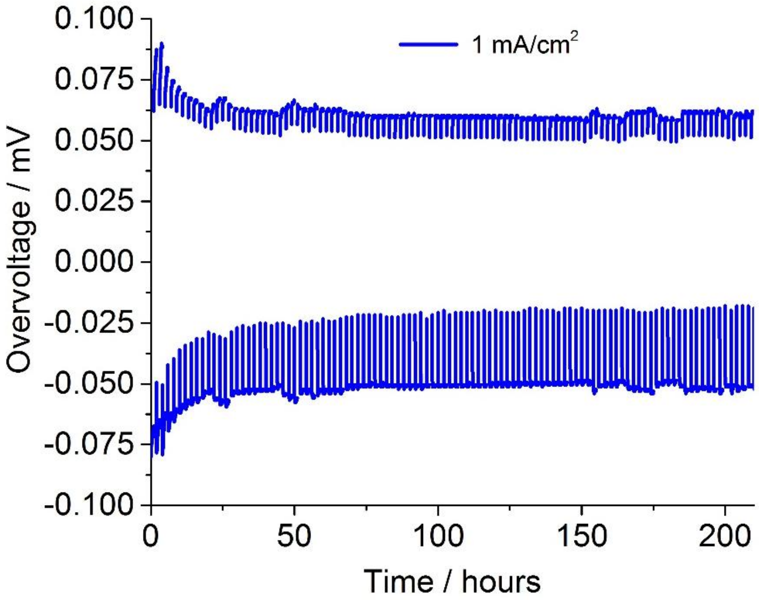

Figure S3. Lithium stripping-deposition overvoltage of the Li-symmetrical cell cycled at a current density of l mA/cm^2 with $LiNO_3$ added to the 0.5M catholyte solution. Current density 1 mA/cm^2 (1h charge/1h discharge)

The morphology of the CNFs materials is characterized by means of Brunauer-Emmet-Teller (BET) method in terms of surface area and pore volume and size distribution. The values are reported and compared in following Table S1.

	**BET surface area (m^2/g)**	**Pore Volume (cm^3/g)**	**Pore size (nm)**
**CNF**	8.3	0.018	34.6
**CNF-KOH**	1313	0.66	3.5
**CNF/SiO_2-KOH-HF**	199	0.48	2.0, 17.9
**CNF/SiO_2-HF-KOH**	924	2.5	3.5, 30.8

Table S1. BET data of the different carbon nano-fibre membranes. Surface area (m^2/g), Pore volume (cm^3/g) and Pore size (nm).

In Figure S4 the capacities of the LiS-cells with different CNF membranes is correlated to the pore volume. The graphic shows how cells with the membrane with smaller pore volume (about 0.02 cm^3/g) also deliver a lower capacity.

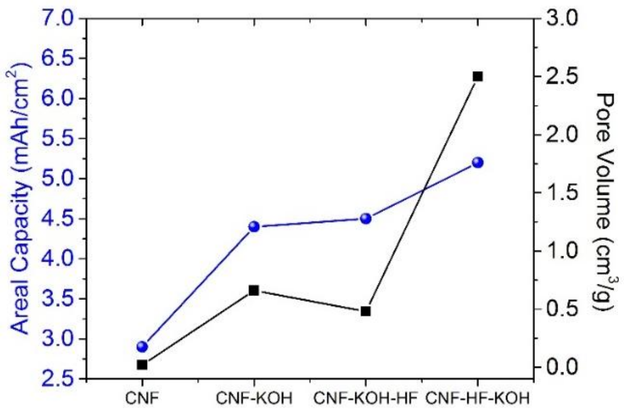

Figure S4 Correlation between the capacity (mAh/cm^2) at 500 μA cm^{-2} current rate of the LiS-cells and the pore volume (cm^3/g) of the CNFs membranes.

TGA analysis has been performed on a **CNF/SiO_2-HF-KOH** membrane after full charge. The data reported in Figure S5 shows firstly the loss of electrolyte, about the 50%, from 25 to 180 °C. The second weight loss between 190 to 270 can be assigned to the sulphur in the meso-pores and on the surface of the CNFs membrane, while the final loss between 280 to 350 °C can be attributed sulphur in the inner pores of the fibres.

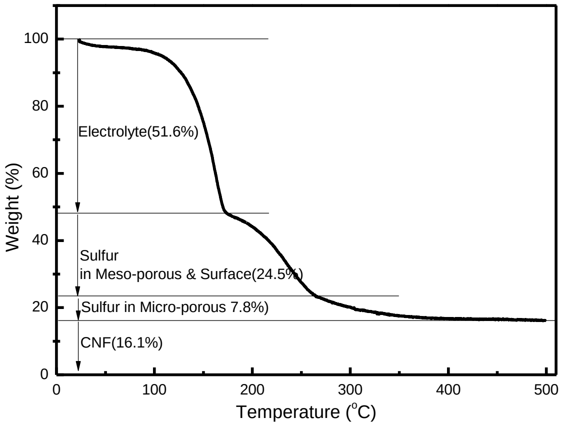

Figure S5. TGA curve of the CNFs membrane after full charge process.

To further study the nature of reaction products after discharge and charge the CNF cathode was immersed in anhydrous ethanol after discharge/charge, see Figure S6. No colour change was found when the charged cathode was soaked in ethanol. This confirms that the lithium polysulphides are completely converted to sulphur, which is not soluble in ethanol [31]. In contrast, the solution containing the discharged cathode changed colour to green, characteristic of the presence of lithium polysulphides.

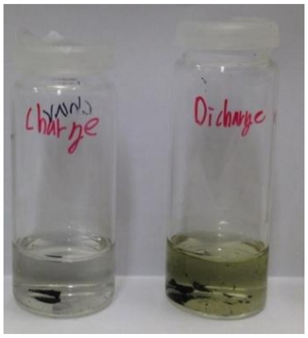

Figure S6. Photograph of CNF/SiO_2-HF-KOH cathode dipped in ethanol after charge/discharge
